# X-ray lasers and serial crystallography

**DOI:** 10.1107/S2052252515008027

**Published:** 2015-04-30

**Authors:** John C. H. Spence

**Affiliations:** a Physics, Arizona State University, Tempe, AZ 85287-1504, USA

**Keywords:** X-ray lasers, serial crystallography

## Abstract

A summary is given of the achievements and opportunities which resulted from the first use of an X-ray laser for serial crystallography and related methods in 2009.

It is now more than five years since protein crystallography data were first collected using a free-electron X-ray laser (XFEL), so it seems an appropriate time for an evaluation of this method of ‘serial femtosecond crystallography’ (SFX) and its relatives.

We have learnt a lot in those five years. Expectations for the first experiments were low, given that the focused beam immediately vaporized the sample, that the pulsed beam, eight orders of magnitude at its peak more intense than a synchrotron, running at 120 Hz at SLAC’s Linac Coherent Light Source (LCLS) facility, had a stochastic wavelength structure, and that shot-to-shot variations in beam intensity were large. Paradoxically, it was found that these hole-drilling femtosecond pulses can outrun radiation damage, so that an almost damage-free diffraction pattern can be recorded before the onset of damage which subsequently vaporizes the sample. And it was found that, since it is applied rapidly, a much larger damage-free dose may be applied than the ‘safe-dose’ limit used at synchrotrons. Then, a complete data set requires a continuous supply of fresh protein microcrystals to be provided, either by scanning fixed-sample mounts or using various types of jet streams. Unlike the 8 Å data collected at 1.8 kV in December 2009 and analysed using millions of diffraction patterns, recent work using a few thousand patterns has yielded structures at better than 2 Å resolution, the result of many incremental improvements in hardware, detectors, software and sample delivery. Viscous jets, for example, have hugely reduced the amount of protein needed (to microgram quantities), while improvements to the original Monte Carlo data analysis method of averaging over all stochastic fluctuations are rapidly appearing, using a range of optimization and iterative methods to implement post-refinement strategies.

The significance of this is that these new structures are obtained at room temperature, without the cooling needed to avoid damage at synchrotrons, thus opening the way for the study of structural dynamics, and that for some proteins (such as the G-protein-coupled receptors), the microcrystals needed may be grown more simply and rapidly than the larger crystals needed at synchrotrons. Where crystal quality rather than damage limits resolution, it remains to be seen whether the use of microcrystals or the sub-micron beam diameter of the LCLS will offer advantages, and much more research is needed to clarify this – here the detailed nature of the crystal defects may be decisive.

But the research opportunities go well beyond these now well established capabilities. The first time-resolved three-dimensional density maps have now been obtained at the LCLS (from Photosystem II and Photoactive yellow protein) using pump–probe methods applied to micro crystals in a liquid jet, and it is clear that the application of new post-refinement methods and viscous jets will soon greatly improve that approach. Other approaches are under rapid development, such as ‘snapshot small-angle X-ray scattering’ or fast solution scattering (FSS) and single-particle imaging, which uses one particle, such as a virus, per shot. Recent time-resolved FSS studies using the pump–probe method have achieved remarkable spatial (*e.g.* 4 Å) and time (sub-picosecond) resolution for proteins whose dark structure is accurately known from previous high-resolution protein crystallography studies. Angular correlation methods may be useful for FSS data with many particles per shot, and are also under development. Mixing jets are planned for dynamic studies, and the integration of microfluidics is well suited to the method of jet sample injection. Finally, the integration of both emission and absorption inner-shell spectroscopies with snap-shot Bragg diffraction is giving us new information on local chemistry in time-resolved experiments, and shows intriguing differences from similar spectra recorded at synchrotrons.

In the National Science Foundation’s ‘BioXFEL’ Science and Technology Center (a consortium of seven US campuses devoted to the use of XFELs for biology – http://www.bioxfel.org) we are often asked when the use of an XFEL may be needed rather than a synchrotron for protein crystallography. Considerations include the following: the need to avoid radiation damage and work at room temperature in a native hydrated environment, the possibility of sub-picosecond time resolution, the ability to study irreversible reactions by time-resolved methods, rather than using stroboscopic methods, the use of microcrystals smaller than the optical absorption length for pump lasers, and small compared with the diffusion length of a substrate, so that fast diffusive mixing is possible in mixing jets. There have been tantalizing suggestions, where comparisons can be made, that, for some proteins, higher resolution is possible at an XFEL; however a comprehensive study which takes crystal quality fully into account remains to be done. Phasing XFEL data by methods other than molecular replacement and isomorphous replacement remains problematic, although several approaches are being actively pursued – single-wavelength anomalous dispersion (SAD) has succeeded in favorable cases, two-color methods which straddle an edge look promising, the use of high beam intensity to ionize sulfur atoms, and so alter their scattering factor, is under development, as is the use of the elastic scattering seen between Bragg reflections in the smallest nanocrystals, due to termination effects.

With new XFELs nearing completion in Switzerland, Germany (Hamburg) and South Korea, the growth of this new field of science seems assured. Since it started soon after LCLS, we have also seen remarkable new research results from the Japanese XFEL (SACLA), paralleling those from the LCLS. Research into much smaller lab-scale XFELs continues. While it is early days for the ‘BioXFEL’ community, it now seems certain that the use of hard X-ray lasers will find its place in structural and dynamic biology, and perhaps now see similar growth to that which occurred when synchrotrons were first applied to this field in the 1970s.

We aim to capture these exciting developments in **IUCrJ**. The journal is now in its second year and has published a number of important X-ray laser and serial crystallography studies so far. A selection of these papers can be found at http://journals.iucr.org/m/services/articles_phys_fels.html.

